# Boron bridging of rhamnogalacturonan‐II is promoted *in vitro* by cationic chaperones, including polyhistidine and wall glycoproteins

**DOI:** 10.1111/nph.13596

**Published:** 2015-08-24

**Authors:** Dimitra Chormova, Stephen C. Fry

**Affiliations:** ^1^ The Edinburgh Cell Wall GroupInstitute of Molecular Plant SciencesSchool of Biological SciencesThe University of EdinburghThe King's BuildingsMayfield RoadEdinburghEH9 3JHUK

**Keywords:** Boron, cell wall, cross‐linking, dehydroascorbic acid, extension, pectic polysaccharides, polyhistidine, rhamnogalacturonan‐II (RG‐II)

## Abstract

- Dimerization of rhamnogalacturonan‐II (RG‐II) via boron cross‐links contributes to the assembly and biophysical properties of the cell wall. Pure RG‐II is efficiently dimerized by boric acid (B(OH)_3_) *in vitro* only if nonbiological agents for example Pb^2+^ are added. By contrast, newly synthesized RG‐II domains dimerize very rapidly *in vivo*. We investigated biological agents that might enable this.
- We tested for three such agents: novel enzymes, borate‐transferring ligands and cationic ‘chaperones’ that facilitate the close approach of two polyanionic RG‐II molecules. Dimerization was monitored electrophoretically.
- Parsley shoot cell‐wall enzymes did not affect RG‐II dimerization *in vitro*. Borate‐binding ligands (apiose, dehydroascorbic acid, alditols) and small organic cations (including polyamines) also lacked consistent effects. Polylysine bound permanently to RG‐II, precluding electrophoretic analysis. However, another polycation, polyhistidine, strongly promoted RG‐II dimerization by B(OH)_3_ without irreversible polyhistidine–RG‐II complexation. Likewise, partially purified spinach extensins (histidine/lysine‐rich cationic glycoproteins), strongly promoted RG‐II dimerization by B(OH)_3_ *in vitro*.
- Thus certain polycations, including polyhistidine and wall glycoproteins, can chaperone RG‐II, manoeuvring this polyanionic polysaccharide domain such that boron‐bridging is favoured. These chaperones dissociate from RG‐II after facilitating its dimerization, indicating that they act catalytically rather than stoichiometrically. We propose a natural role for extensin–RG‐II interaction in steering cell‐wall assembly.

## Introduction

Healthy plant growth and development are dependent on an appropriate soil concentration of soluble boric acid (B(OH)_3_) (Blevins & Lukaszewski, 1998; Goldbach & Wimmer, 2007). Although boron (B) deficiency can often be solved by the application of fertilizers, excess boron is an intractable agricultural problem, and there is a narrow concentration‐range between boron deficiency and excess (Reid *et al*., 2004). Exploring the roles and behaviour of boron in plants may lead to advances with both pure and applied interest.

Adequate boron is essential throughout the plant, but particular roles exist in pollen‐tube and root‐nodule development (Bolaños *et al*., 2004). Higher boron is required when excess Al^3+^ is present, usually in acidic soils; and sandy or chalky soils are often deficient in available boron (Shorrocks, 1997). Insoluble oxides and hydroxides such as those of aluminium (Al) and iron (Fe) act as boron adsorbing surfaces in soils, and affect its behaviour and availability (Goldberg, 1997). Some of the potentially available boron in soil is adsorbed on organic ligands, from which it is gradually released as available B(OH)_3_, a Lewis acid which forms the borate anion only at high pH:

$${\text{B(OH)}}_{3}+H_{2}O↔{\text{B(OH)}}_{4}^{-}+H^{+}\;\;\left({\text{pK}}_{a}\approx 9.1\right).$$

The best‐established role of boron in plants is as a covalent bridge between pectin molecules (O'Neill *et al*., 1996). Such bridging decreases wall porosity (Fleischer *et al*., 1998, 1999) and modifies the wall's biomechanical properties, thickness and growth (Hirsch & Torrey, 1980; Hu & Brown, 1994; Findeklee & Goldbach, 1996; Ishii *et al*., 2001). Pectin‐rich tissues (e.g. collenchyma) show especially striking deficiency symptoms, and boron requirements of different plants correlate with their pectin content (Hu *et al*., 1996). Loomis and Durst (1992) first suggested that apiose, a component of pectin, is the key wall residue to which boron binds. Pectins are galacturonate‐rich polysaccharides, built of up to four domains (homogalacturonan, rhamnogalacturonan‐I (RG‐I), RG‐II and xylogalacturonan), which are glycosidically inter‐linked (Ishii *et al*., 2001; Coenen *et al*., 2007; Albersheim *et al*., 2011). Boron specifically binds to RG‐II, a small (degree of polymerization (DP) typically 29–30; i.e. *c*. 5 kDa) but complex, taxonomically conservative pectic domain. To RG‐II's acidic backbone of about eight (1→4)‐α‐galacturonate residues (identical to, and probably contiguous with, homogalacturonan) are attached five unique side‐chains, A–E. Boron avidly binds to the β‐d‐apiose residue of side‐chain A, which also possesses residues of α‐l‐galactose, β‐d‐glucuronate (and/or its methyl ester), 2‐*O*‐methyl α‐d‐xylose, α‐l‐fucose, β‐l‐rhamnose, α‐d‐galacturonate and β‐d‐galacturonate (and/or its methyl ethers) (O'Neill *et al*., 2004; Pabst *et al*., 2013). The formation of RG‐II–(B^−^)–RG‐II bridges is a major reason why plants require boron, and why the pectin‐poor Poales need less boron than dicots.

Little is known about the mechanisms of the boron bridging of RG‐II, especially under biologically relevant (e.g. Pb^2+^‐free) conditions. Many neutral sugars rapidly esterify with borate at pH *c*. 9 to form negatively charged esters (a fact exploited in the electrophoresis of otherwise neutral sugars; Weigel, 1963; Goubet *et al*., 2006), but the bonds formed are unstable at pH values (< 7) characteristic of the cell wall. They are thus not valid models of B–RG‐II bridging. Furanosyl *cis*‐1,2‐diols (e.g. ribofuranose in NAD^+^; apiofuranose in methyl β‐apioside; and hydrated 1‐deoxy‐3‐keto‐l‐ribulose, a quorum‐sensing bacterial metabolite; Chen *et al*., 2002) form B‐esters that are more stable than their *trans*‐diol or pyranosyl counterparts (Ishii & Ono, 1999), but even these are weak and transient compared with B–RG‐II bridges. The latter are stable enough to withstand column chromatography in or dialysis against mildly acidic, B‐free buffers; for example, their half‐life at pH 2.8 and 20°C is *c*. 24 h (O'Neill *et al*., 1996).

While slow to break, B–RG‐II bridges are also slow to form *in vitro* when pure RG‐II is mixed with B(OH)_3_. Such bridging is somewhat promoted by high (e.g. 50 mM) Ca^2+^ at pH 5.0, and more so at pH 3.5 (O'Neill *et al*., 1996; Ishii & Ono, 1999). In addition, some nonbiological cations (e.g. 0.5 mM Pb^2+^ or Sr^2+^) strongly enhance RG‐II bridging by B(OH)_3_ *in vitro* (O'Neill *et al*., 1996; Ishii & Ono, 1999). The more biologically relevant Ca^2+^ ion is effective only at much higher concentrations, for example 50 mM or at low pH values such as 3.5 (Ishii *et al*., 1999). It remains unknown what biological agent ‘replaces’ Pb^2+^ etc. *in vivo*.

**Figure 1:**
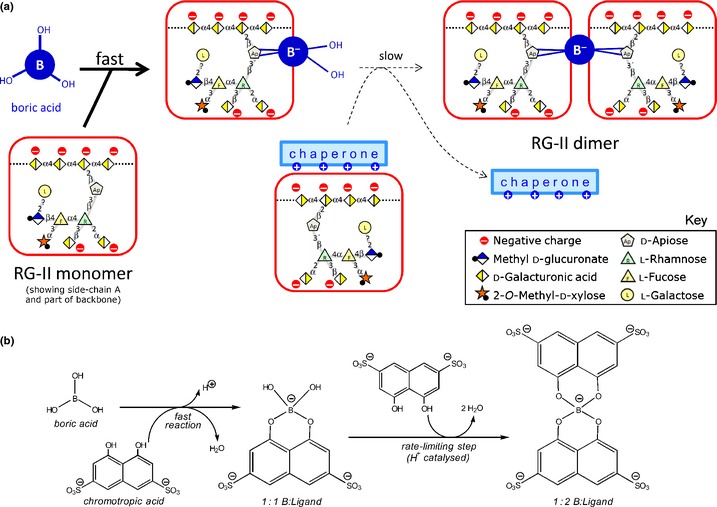
Proposed two‐step dimerization of polyanionic rhamnogalacturonan‐II (RG‐II) and of chromotropic acid as a model. (a) The polyanion, RG‐II ^(n−)^, is proposed to undergo two reactions similar to those shown in (b). Owing to the strong negative charge on both RG‐II molecules, we propose that the second step will be favoured if the RG‐II ^(n−)^ is ionically complexed with a cationic ‘chaperone’. (b) The ionized form of chromotropic acid (CTA ^2−^) rapidly reacts with neutral trigonal boric acid (B(OH)_3_), giving a (B:CTA)^3−^ complex containing a tetrahedral B^−^. This reacts slowly with a second CTA ^2−^, despite electrostatic repulsion, to give a relatively stable (B:(CTA)_2_)^5−^ complex (Shao *et al*., 2000).

Un‐ionized B(OH)_3_ probably binds quickly but reversibly to the side‐chain A apiofuranose residue of a monomeric RG‐II domain, forming a negatively charged apiose–B^(−)^(OH)_2_ ester (Fig. 1a). However, in addition to the negative charge on the boron atom, this apiose residue is surrounded by several negatively charged sugar residues, all of which will tend to repel an incoming second RG‐II domain, thus hindering dimerization. A comparable two‐stage boron‐bridging process has been described for chromotropic acid (CTA; Fig. 1b) (Shao *et al*., 2000), and the formation of the 1 : 2 (CTA–B^(−)^–CTA) complex may be a helpful model for studies of RG‐II dimerization. Interestingly, negatively charged groups neighbouring the boron atom can stabilize B‐bridges once formed, as shown in ^11^B‐NMR studies of CTA (Shao *et al*., 2000). As with RG‐II bridging, CTA bridging is promoted by H^+^ and is undetectable above pH 7–8. At equilibrium at the optimal pH (4.5), *c*. 70% of the CTA is in the CTA–B^(−)^–CTA complex (Shao *et al*., 2000). Both the formation and the hydrolysis of the CTA complex are promoted by low pH, as in the case of the RG‐II complex (O'Neill *et al*., 2004).

We recently defined the kinetics of B‐bridge formation between RG‐II domains *in vivo* (Chormova *et al*., 2014a). We developed a convenient polyacrylamide gel‐electrophoresis (PAGE) method for separating monomeric from dimeric (boron‐bridged) RG‐II, and used it to confirm that Pb^2+^ promotes the B(OH)_3_‐dependent dimerization *in vitro*, previously reported by O'Neill *et al*. (1996) using alternative techniques. For *in vivo* experiments, we successfully cultured ‘Paul's Scarlet’ rose (*Rosa* sp.) cells in a boron‐free medium: their wall‐bound pectin contained monomeric RG‐II domains and no detectable dimers. Readdition of 3.3 μM B(OH)_3_ to these cultures triggered a gradual production of RG‐II dimer over 24 h, but without any detectable loss of existing monomers, suggesting that only new RG‐II domains, synthesized after the addition of boron, were amenable to boron‐bridging (Chormova *et al*., 2014a). In agreement with this, boron‐starved *Rosa* cultures whose biosynthetic machinery had been compromised (by carbon starvation, respiratory inhibitors, anaerobiosis, freezing or boiling) lost the ability to generate RG‐II dimers in response to boron readdition. We concluded that RG‐II normally becomes boron‐bridged during synthesis (within the Golgi system) or during subsequent secretion (across the plasma membrane), but not post‐secretion. Supporting this conclusion, exogenous radioactive RG‐II neither dimerized in the medium nor became cross‐linked to existing cell‐wall RG‐II domains (Chormova *et al*., 2014a). Thus, in cultured *Rosa* cells, RG‐II domains have a brief window of opportunity for boron‐bridging, intraprotoplasmically and/or during secretion; however, secretion into the apoplast is a point of no return beyond which additional boron‐bridging does not readily occur (Chormova *et al*., 2014b).

Previous results suggested rapid dimerization of pre‐existing monomeric RG‐II *in vivo* since the addition of B(OH)_3_ to living plant material had rapid effects on wall pore size (Fleischer *et al*., 1999) and on wall thickness (Ishii *et al*., 2001). However, based on recent findings (Chormova *et al*., 2014a,b), pre‐existing monomeric RG‐II does not rapidly dimerize in response to added B(OH)_3_ *in vivo*. The assumption that boron affects the physical properties of the cell wall only by interacting with RG‐II (and thus that the effects of added boron are necessarily due to RG‐II dimerization) might be false: among other possibilities, boron may also influence wall–membrane interactions (Voxeur & Fry, 2014).

To build on our *in vivo* findings, we have now applied the PAGE method to assay RG‐II dimerization *in vitro*, with the goal of defining the naturally occurring components, besides B(OH)_3_ and free RG‐II, that facilitate the formation of boron bridges and thus effect under biologically relevant conditions what Pb^2+^ ions have been shown to achieve. Theoretical possibilities include a novel boron‐acting enzyme, a cationic chaperone of RG‐II or a boryl‐transfer ligand.

### Possible novel enzyme

O'Neill *et al*. (1996) speculated that the making and breaking of RG‐II–B^(−)^–RG‐II bridges *in vivo* is enzymic, but no evidence for such enzymes yet exists.

### Cationic chaperones

A suitably sited Pb^2+^ ion facilitates RG‐II dimerization *in vitro*, possibly by neutralizing negative charges on the anionic RG‐II (Fig. 1a; where ‘chaperone’ = Pb^2+^). This cannot be the precise mechanism *in vivo* since lead (Pb) is not an essential element for plants. Nevertheless, it is very unlikely that free RG‐II, which is routinely used for *in vitro* cross‐linking studies, is the natural substrate during B‐bridging. Instead, the biologically relevant pectic substrate for B‐bridging *in vivo* is likely to be the RG‐II domains that are ionically complexed with cations within the Golgi system. Biologically significant cations besides Ca^2+^ in the appropriate subcellular location could include polyamines and basic glycoproteins such as extensins. Indeed, extensins, like pectins and the requirement for boron, are minimal in the Poales. Thus, RG‐II could be chaperoned by organic cations to steer B‐bridging in a way that is not achievable by pure RG‐II *in vitro*.

### Boron ‘donor substrates’

Another uncertainty concerning the *in vivo* dimerization of RG‐II is the chemical form of the supplied boron. The bridging reactions may proceed as in Fig. 1(a), starting with free B(OH)_3_. Indeed, most of the soluble boron in plants is apoplastic B(OH)_3_ (Matoh, 1997) except in high‐pH phloem sap (Hu *et al*., 1997). But an alternative possibility is that B(OH)_3_ first complexes with a nonpectic ligand (L; presumably a diol), which then acts as a ‘donor substrate’ to transfer the boron to RG‐II (Voxeur & Fry, 2014). According to this hypothesis, L would promote RG‐II dimerization in a series of reactions such as:

$$\begin{array}{cc} L\cdot {\left(\text{OH}\right)}_{2} & +{\text{B(OH)}}_{3}\\ & ↔L\cdot {\left({\text{BO}}_{4}H_{2}\right)}^{-}+H_{2}O+H^{+}\;\;\left(\text{Reaction 1}\right) \end{array}$$

$$\begin{array}{cc} L\cdot {\left({\text{BO}}_{4}H_{2}\right)}^{-} & +\text{RG‐II}\cdot {\left(\text{OH}\right)}_{2}\\ & ↔L\cdot {\left({\text{BO}}_{4}\right)}^{-}\cdot \text{RG‐II}+2H_{2}O\;\;\left(\text{Reaction 2}\right) \end{array}$$

$$\begin{array}{cc} L\cdot {\left({\text{BO}}_{4}\right)}^{-}\cdot \text{RG‐II} & +\text{RG‐II}\cdot {\left(\text{OH}\right)}_{2}\\ & ↔L\cdot {\left(\text{OH}\right)}_{2}+\text{RG‐II}\cdot {\left({\text{BO}}_{4}\right)}^{-}\cdot \text{RG‐II}\;\;\left(\text{Reaction 3}\right) \end{array}$$

In reaction 1, boric acid binds to L; in reaction 2, L forms a B‐centred complex with RG‐II; and finally in reaction 3, L is displaced by an incoming second RG‐II molecule, resulting in a firmly cross‐linked RG‐II–B–RG‐II dimer. One or more of these reactions could be enzyme‐catalysed.

The ligand L could be a membrane‐bound glycosylinositol phosphorylceramide (Voxeur & Fry, 2014) or an apoplastic solute such as a sugar, alditol, cyclitol or ascorbate metabolite. Boron complexation by dehydroascorbic acid has been reported (Polle *et al*., 1990), but little studied. In this connection, it is interesting that root growth can be maintained in very low‐boron media if the latter are supplemented with ascorbate (Lukaszewski & Blevins, 1996) (which yields several apoplastic oxidation products *in vivo*; Green & Fry, 2005; Parsons *et al*., 2011; Parsons & Fry, 2012), suggesting that ascorbate or its metabolites can enhance the effectiveness of boron in plants.

The main objectives of this work, inspired by the foregoing background, were: testing the possible action of novel enzymes in borate bridging, identifying natural RG‐II chaperones which would facilitate the close approach of two otherwise mutually repulsive RG‐II domains to facilitate B–RG‐II bridging, and identifying possible borate ‘donor’ substrates involved in B–RG‐II bridging.

## Materials and Methods

### Materials

Poly‐l‐lysine (Br^−^; mean DP by viscometry 115), poly‐l‐arginine (Cl^−^; DP 61), poly‐l‐histidine (Cl^−^; DP 106), poly‐l‐glutamate (Na^+^; DP 240), boric acid, lead nitrate, l‐ascorbic acid, dehydro‐l‐ascorbic acid, sugars, alditols, *myo*‐inositol, polyamines, l‐lysine and buffer components were all from Sigma–Aldrich (Dorset, UK).

### Extraction of parsley enzymes

Proteins were extracted from parsley (*Petroselinum crispum*) shoots in 0.2 M succinate (Na^+^) buffer, pH 5.5, as described (Franková & Fry, 2011). Enzyme activities present in this extract are catalogued in *GHATA*base (http://fry.bio.ed.ac.uk//GHATAbase.html).

### Rhamnogalacturonan‐II

RG‐II was isolated from cultured *Arabidopsis thaliana* or *Rosa* cells by treatment with Na_2_CO_3_ followed by Megazyme endopolygalacturonase, purified by gel‐permeation chromatography on Bio‐Gel P‐30 monomerized with cold 0.125 M HCl, and freed of acid on Bio‐Gel P‐2, as described by Chormova *et al*. (2014a). Radiolabelled monomeric RG‐II (17 MBq μmol^−1^) was prepared as described (Chormova *et al*., 2014a).

### Gel electrophoresis

Monomeric and dimeric RG‐II were separated by PAGE and either stained for polysaccharide with a silver reagent or fluorographed for tritium (followed by quantification by scintillation counting), all as described by Chormova *et al*. (2014a). Briefly, 8 μl samples were mixed with 2 μl of sample buffer and subjected to electrophoresis at 200 V for 75 min.

### Isolation and fractionation of extensin

Extensin was solubilized from the surface of living suspension‐cultured spinach cells with 0.1 M CaCl_2_, dialysed against 1 mM mercaptoethanol, and freeze‐dried as described by Miller and Fry (1992). Samples of the extensin preparation were checked by sodium dodecyl sulphate (SDS)–PAGE in a 15% gel and stained with Coomassie Brilliant Blue or periodate/Schiff reagent (Matthieu & Quarles, 1973). Markers included potato lectin, and de‐arabinofuranosylated potato lectin, kindly provided by Dr R. J. Owens, University of Cambridge (Owens & Northcote, 1980). A further sample of an identical but radiolabelled extensin preparation (obtained from a spinach culture that had been incubated in the presence of a trace of [^3^H]arabinose for 6 d; Fry & Northcote, 1983) was checked for hydroxyproline oligoarabinoside residues: the [^3^H]extensin was incubated in 0.2 M Ba(OH)_2_ at 110°C for 16 h, Ba^2+^ was removed as its insoluble sulphate after buffering to pH 2 with H_2_SO_4_, and the soluble products were analysed for (positively charged) hydroxyproline oligo‐[^3^H]arabinosides by paper electrophoresis at pH 2 and 5 kV for 30 min (Fry, 2011). Strips of the electrophoretogram were assayed for radioactivity by scintillation counting.

To monitor the provenance of the eluted extensin, we studied its appearance in and disappearance from growing cells. Spinach cell cultures were harvested at intervals after subculturing, their spent medium was removed, and the cells were freeze‐dried; total cellular protein was then extracted from the cells with shaking in phenol : acetic acid : water (2 : 1 : 1, w/v/v; 0.1 ml mg^−1^ DW) at 53°C for 4 h. Proteins were precipitated from 500 μl of the extract by addition of 10 μl 10% (w/v, aqueous) ammonium formate followed by 2.5 ml acetone and storage at 4°C for 16 h. The protein pellet was washed in 80% acetone, dried and redissolved in Laemmli sample buffer. A portion (equivalent to 0.8 mg DW of cells) was run by SDS‐PAGE in a 15% gel and stained with Coomassie Brilliant Blue.

The crude extensin preparation was fractionated by cation‐exchange chromatography on Sulphopropyl (SP)‐Sephadex C‐25 (from Pharmacia Fine Chemicals, Uppsala, Sweden; currently available from GE Healthcare Life Sciences, Little Chalfont, UK; http://www.gelifesciences.com/) as described by Biggs and Fry (1990). Buffers for cation‐exchange chromatography all contained 0.1 M Na^+^ ions (added as NaOH) and were adjusted to the required pH with solid HEPES (pH 7.5 and 8.0) or TAPS (pH 8.5 and 9.0) or with 0.1 M NaHCO_3_ (to pH 9.5–10.5). Extensin (3 mg) was dissolved in 1 ml of the pH 7.5 buffer and passed through a 5‐ml bed‐volume column of SP‐Sephadex (Na^+^ form, pre‐equilibrated with the same buffer) at 0.2 ml min^−1^. Ten millilitres of each buffer was then passed through the column at 0.2 ml min^−1^, and 2‐ml fractions were collected. Appropriate fractions (as judged by *A*_280_) were combined into six pools, each of which was dialysed against 1 mM mercaptoethanol, dried and redissolved in 200 μl water.

### Assays of RG‐II dimerization

Monomerized RG‐II (final concentration *c*. 100 μg ml^−1^ (*c*. 20 μM) nonradioactive RG‐II, or 300 kBq ml^−1^ (*c*. 18 μM) [^3^H]RG‐II) in 50 mM acetate (Na^+^) buffer, pH 4.8, was incubated for 3–24 h in the presence of the reagents and polypeptides indicated in individual experiments. Pb^2+^ was supplied as Pb(NO_3_)_2_. The results were monitored by electrophoresis of an 8‐μl sample containing *c*. 0.8 μg or 2.4 kBq of RG‐II.

## Results

### The possibility of a borate esterase activity in plant extracts

**Figure 2:**
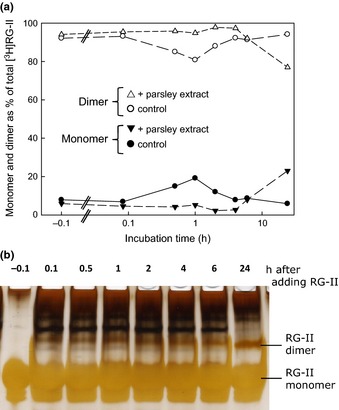
A parsley extract rich in numerous wall‐modifying enzyme activities fails to dimerize rhamnogalacturonan‐II (RG‐II). Monomeric RG‐II (a, 18 μM tritiated; b, 20 μM nonradioactive) was incubated in 1.2 mM B(OH)_3_ at 20°C in the presence or absence of a wall‐enzyme‐rich extract from parsley shoots for 0–24 h, then frozen at −80°C. Later, (a) 2.4 kBq or (b) 0.8 μg of the RG‐II was subjected to polyacrylamide gel electrophoresis. In (b), the gel was silver‐stained and photographed. In (a), the zones corresponding to monomer and dimer were excised from the gel and separately assayed for radioactivity by scintillation counting. The ‘−0.1 h’ sample was taken just before addition of the parsley extract.

Given that no boron‐metabolizing enzyme is yet known, we undertook a preliminary study of the possibility that a plant protein might catalyse the boron‐dependent dimerization of RG‐II, thus ‘mimicking’ the action of Pb^2+^. The potential source was an extract of parsley shoots, a proven rich source of numerous cell‐wall‐directed enzyme activities including xyloglucan endotransglucosylase (XET), glycosidases, glycanases, transglycosidases, transglycanases and pectin methylesterase (*GHATA*base, 2015; Franková & Fry, 2011, 2012, 2013; D. Chormova & S. C. Fry, unpublished). When the parsley extract was incubated for 0–24 h with [^3^H]RG‐II (Fig. 2a) or with nonradioactive RG‐II (Fig. 2b), plus B(OH)_3_, *in vitro*, we found negligible dimerization compared with that caused by Pb^2+^. There was thus no positive evidence for a borate esterase or boryltransferase under the conditions used.

### Potential borate donor substrates

**Figure 3:**
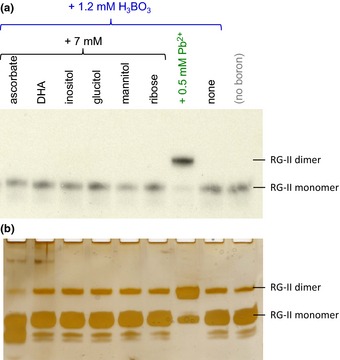
Low‐*M*_r_ putative boron carriers do not promote rhamnogalacturonan‐II (RG‐II) dimerization. Monomeric RG‐II (a, 18 μM tritiated; b, 20 μM nonradioactive) was incubated at 20°C for 24 h in the presence of the additives indicated at the top. DHA, dehydroascorbic acid. Later, (a) 2.4 kBq or (b) 0.8 μg of the RG‐II was subjected to polyacrylamide gel electrophoresis. In (a) the radioactive bands on the gel were detected by fluorography; in (b), the gel was silver‐stained and photographed.

**Figure 4:**
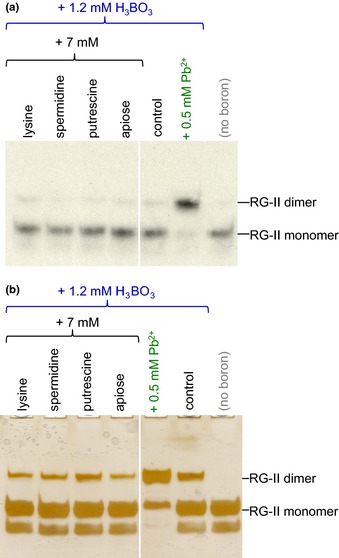
Low‐*M*_r_ organic cations and free apiose do not promote rhamnogalacturonan‐II (RG‐II) dimerization. Monomeric RG‐II (a, 18 μM tritiated; b, 20 μM nonradioactive) was incubated at 20°C for 24 h in the presence of the additives indicated at the top. Later, (a) 2.4 kBq or (b) 0.8 μg of the RG‐II was subjected to polyacrylamide gel electrophoresis. In (a) the radioactive bands on the gel were detected by fluorography; in (b), the gel was silver‐stained and photographed.

We tested the hypothesis that compounds with an affinity for B(OH)_3_ might function as a borate ‘donor‐substrate’, transferring boron to an RG‐II molecule, and thus promoting dimerization. Compounds tested (Figs 3, 4) included sugars that readily form furanose *cis*‐diols (ribose and apiose), two alditols (glucitol, mannitol), a cyclitol (inositol), ascorbate and its initial oxidation product (dehydroascorbic acid). None of these compounds promoted the *in vitro* dimerization of RG‐II. In some experiments (e.g. Fig. 3b), ascorbic acid inhibited dimerization.

### Cations as chaperones of RG‐II

In view of the known effects of some inorganic cations (e.g. Ca^2+^ and Pb^2+^), we tested whether adding small *organic* cations might facilitate the cross‐linking of the polyanion RG‐II (Fig. 4). The compounds tested were two polyamines (putrescine and spermidine) and lysine, none of which appreciably affected the dimerization of either ^3^H‐labelled or pure nonradioactive RG‐II.

By contrast, a promotion of RG‐II dimerization was evoked by certain large organic cations. At 1 mg ml^−1^, polyarginine (and, as expected, anionic polyglutamate) had no discernible effect on RG‐II (Supporting Information Fig. S1). Polylysine caused the complete disappearance of RG‐II from the gel electrophoretogram, probably because it formed a stable polylysine–RG‐II ionic complex with a net positive charge and thus migrated towards the cathode rather than into the gel. Polyhistidine had an intermediate behaviour: it favoured RG‐II dimerization about as effectively as Pb^2+^ (Fig. S1), and both the RG‐II dimer and the remaining monomer had the same electrophoretic mobility as in the absence of polycations, indicating that the polyhistidine–RG‐II ionic complex dissociated during electrophoresis.

**Figure 5:**
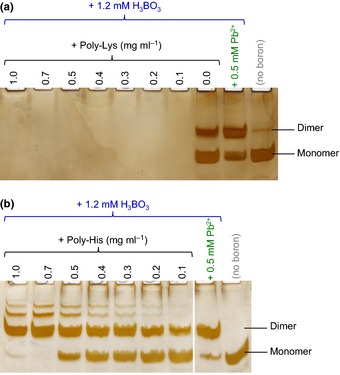
Polylysine and polyhistidine have different effects on rhamnogalacturonan‐II (RG‐II). Monomeric RG‐II (0.1 mg ml^−1^; 20 μM) was incubated at 20°C for 24 h with a dilution series of (a) polylysine and (b) polyhistidine, with and without the other additives indicated. Later, 0.8 μg of the RG‐II was subjected to polyacrylamide gel electrophoresis, and the gel was silver‐stained and photographed.

A dilution series showed that relatively low concentrations of polyhistidine and polylysine, as low as 0.1 mg ml^−1^, had the above‐noted effects (Fig. 5). This concentration is equal to that of the monomeric RG‐II present in the reaction mixture.

### Preparation and partial characterization of spinach extensin

Plants possess a range of polycations, including histidine‐ and lysine‐rich (glyco)proteins (Cassab *et al*., 1985; Kieliszewski *et al*., 1992; Sun *et al*., 2005). Many extensins are rich in both histidine and lysine residues, and we therefore wished to test an extensin preparation for biologically relevant RG‐II chaperoning effects. We used CaCl_2_ to solubilize ionically wall‐bound extensin from live spinach cells by a procedure reported before (Miller & Fry, 1992). The product's amino‐ and imino‐acid composition includes 31.8 mol% hydroxyproline, 6.3 mol% His and 7.8 mol% Lys, but only 2.4 mol% (Glu + Gln) and 0.7 mol% (Asp + Asn), and this extensin is thus highly basic (Biggs & Fry, 1990).

**Figure 6:**
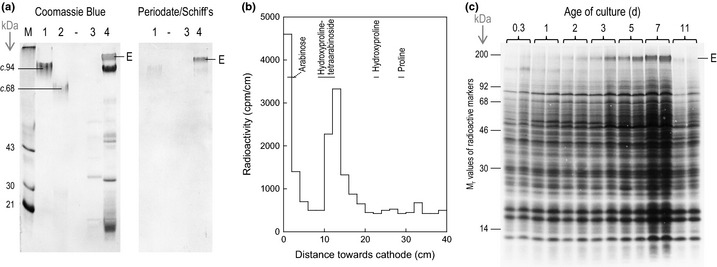
Partial characterization of a spinach extensin preparation. (a) Sodium dodecyl sulphate–polyacrylamide gel electrophoresis (SDS‐PAGE) of (glyco)proteins eluted from the cells of a live 6‐d‐old spinach suspension‐culture with water or 0.1 M CaCl_2_, and comparable markers. M, commercial protein marker‐mixture. Lanes: 1, potato lectin; 2, de‐arabinofuranosylated potato lectin; 3, water‐eluate of spinach cells; 4, 0.1 M CaCl_2_‐eluate of spinach cells; ‐, blank lane. Stains: left panel, Coomassie Blue (showing total proteins); right panel, periodate/Schiff's reagent (showing glycoproteins). E, spinach extensin. (b) High‐voltage paper electrophoresis (at pH 2.0) of the alkali hydrolysis products of [*pentosyl*‐^3^H]extensin. Nonradioactive markers were run in parallel and detected by staining (horizontal lines). (c) SDS‐PAGE of total cellular proteins extracted with warm phenol/acetic acid/water from suspension‐cultured spinach cells at various times (0.3–11 d after subculture). Stain: Coomassie Blue. E, spinach extensin. Each loading is the equivalent of 0.8 mg DW of cells.

We showed here that the CaCl_2_‐eluted preparation contained an abundant high‐*M*_r_ protein (*M*_r_ > 100 000 when compared with potato lectin; Owens & Northcote, 1980), which stained strongly with periodate/Schiff's reagent, indicating that it was a glycoprotein (band ‘E’ in Fig. 6a). With Coomassie Blue, it stained a characteristic magenta colour easily distinguishable from the usual blue of other proteins, probably because of its high positive charge. A comparable extensin preparation eluted from [^3^H]arabinose‐fed spinach cells yielded a peak of hydroxyproline tetra‐[^3^H]arabinoside on alkaline hydrolysis (Fig. 6b). A similar high‐*M*_r_ glycoprotein showed up as a major band among the total phenol‐extractable cellular (protoplast + wall) proteins; its abundance steadily increased from 0.3 to 7 d after subculturing, but then suddenly decreased (Fig. 6c) – possibly owing to tyrosine‐based covalent cross‐linking (Brady & Fry, 1997). All these observations support the conclusion that the CaCl_2_‐eluted preparation was rich in a typical extensin.

### Extensin as a natural cationic chaperone of RG‐II

**Figure 7:**
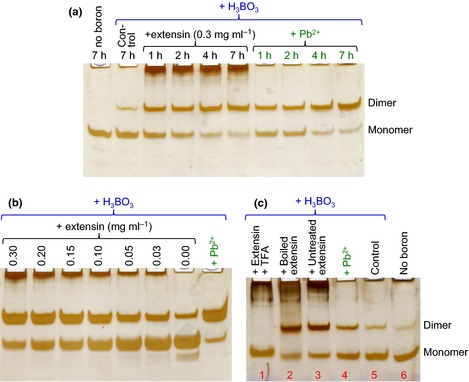
An extensin preparation promotes rhamnogalacturonan‐II (RG‐II) dimerization. Monomeric RG‐II (0.1 mg ml^−1^; 20 μM) was incubated at 20°C in the presence of various combinations of 1.2 mM boric acid, 0.5 mM Pb^2+^ and crude spinach extensin. In (a), the incubation time was varied. In (b), the extensin concentration was varied, incubation time being held constant at 4 h. In (c), the extensin, when present, was at a final concentration of 0.3 mg ml^−1^, and the incubation time was 3 h; the extensin used for the sample in lane 2 had been pretreated at 100°C for 30 min before being mixed with RG‐II; that for lane 3 was an unboiled control; and in lane 1 the reaction was conducted in the presence of nonboiled extensin plus 0.2 M trifluoroacetic acid (TFA). In each case, 0.8 μg of the RG‐II was subjected to polyacrylamide gel electrophoresis, and the gel was then silver‐stained and photographed.

The soluble spinach extensin preparation, at 0.03–0.3 mg ml^−1^, ‘mimicked’ Pb^2+^ and polyhistidine in promoting RG‐II dimerization; the effect, like that of Pb^2+^, was readily detectable within 1 h (Fig. 7a), and was concentration‐dependent (Fig. 7b). Detection of the effect within 1 h compares favourably with the incubation times (*c*. 24 h) typically used *in vitro* for demonstrating the effects of Pb^2+^ (O'Neill *et al*., 1996). The effectiveness of the extensin was not compromised by boiling (Fig. 7c), indicating that it was not acting enzymically, but physically – presumably as a chaperone of the polyanionic RG‐II. The presence of 200 mM trifluoroacetic acid (an excess over the 50 mM acetate buffer present) during the 3 h incubation of RG‐II with extensin blocked the dimerization (Fig. 7c).

**Figure 8:**
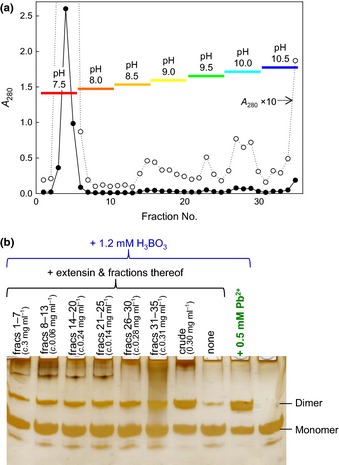
Cation‐exchange fractions of crude extensin promote rhamnogalacturonan‐II (RG‐II) dimerization. (a) Fractionation of 3 mg crude spinach extensin on sulphopropyl‐Sephadex (SP‐Sephadex) by elution with a pH gradient. Column bed volume, 5 ml; fraction volume, 2 ml. Bars indicate the pH of the eluent being applied to the column while the indicated fractions were being collected. (b) Monomeric RG‐II (0.1 mg ml^−1^; 20 μM) was incubated at 20°C for 4 h with the (dialysed and dried) extensin fractions and/or other additives as indicated. Approximate concentrations of the extensin fractions present in the reaction mixtures were estimated from *A*_280_ (each concentration is the equivalent of the whole pool diluted into 2 ml of reaction mixture). After the incubation, 0.8 μg of the RG‐II was subjected to polyacrylamide gel electrophoresis, and the gel was then silver‐stained and photographed.

Cation‐exchange chromatography of the extensin gave fractions that eluted as the pH was raised from 7.5 to 10.5 (Fig. 8a). After dialysis and drying, each pool was tested for its effect on RG‐II dimerization. Each pool caused more dimerization than the extensin‐free control (Fig. 8b). No pool was as effective as 0.3 mg ml^−1^ crude extensin preparation, suggesting that a minor subpopulation of the extensin was most effective.

## Discussion

In living plant cells, newly synthesized RG‐II is rapidly dimerized before and/or during secretion into the cell wall. This process cannot readily be resumed once the RG‐II has become integrated into the wall, nor can it be mimicked if free RG‐II is added to the medium of cultured plant cells (Chormova *et al*., 2014a). In the absence of living plant cells, dimerization *in vitro* is very slow unless nonbiological cations such as Pb^2+^, Sr^2+^ or very high Ca^2+^ are added (O'Neill *et al*., 1996). These observations strongly suggest that unique features of the local environment within the Golgi/secretion system *in vivo* favour the dimerization of RG‐II. We investigated the possible nature of the proposed biological agents that may thus promote RG‐II dimerization. Theoretical possibilities included a novel enzyme, a boryl‐transfer ligand or a cationic chaperone of RG‐II.

The results presented in this manuscript are qualitative rather than quantitative; however, all gels included simultaneously run controls, and the electrophoretic patterns shown are representative of at least three replicate experiments. We report that neither a parsley shoot enzyme extract (rich in wall‐modifying enzymes) nor a range of potential boron carriers (e.g. dehydroascorbic acid) was able to promote RG‐II dimerization *in vitro*. Thus, we obtained no positive evidence for a novel borate esterase or boryltransferase activity, nor for a boryl‐transferring ligand. However, absence of evidence is not evidence of absence, and additional enzyme extracts and low‐*M*_r_ compounds should still be tested. Also, it remains possible that a boron carrier *plus* an enzyme might favour RG‐II dimerization. Further experiments are required to investigate the wide range of possibilities: the PAGE approach used here clearly provides a convenient methodological base for such future studies.

Positive results were obtained in our screen for organic cationic ‘chaperones’ that promote RG‐II dimerization. Although small organic cations (spermidine, putrescine and lysine) did not enhance dimerization, some cationic polymers did. In a comparison of three positively charged poly(amino acids), major differences were noted between polylysine, polyarginine and polyhistidine, even though the side‐chains of all three of these would be cationic at the pH (4.8) used in boron‐bridging experiments (approximate side‐chain pK_a_ values being: lysine, 10.5; arginine, 12.5; histidine, 6.0), and all three poly(amino acids) would be expected to adopt random‐coil conformations owing to mutual repulsion of the pendant side‐chains. Polylysine, with each side‐chain bearing a full positive charge on its single ε‐N atom, evidently bound to RG‐II to form an ionic complex with a net positive charge and with a sufficient longevity that none of the RG‐II was able to electrophorese towards the anode (Fig. 5a). Polyarginine, each side‐chain bearing a full positive charge that is, however, delocalized between three N atoms, had no perceptible effect on the electrophoretic behaviour of RG‐II or on its ability to dimerize. However, polyhistidine, the least fully ionized of the three poly(amino acids) tested, evidently had an appropriate charge density such that it enabled the close approach and dimerization of two RG‐II molecules but also subsequently released them, as evidenced by the normal electrophoretic behaviour of the monomeric and dimeric RG‐II; Pb^2+^‐generated dimeric RG‐II was indistinguishable from the polyhistidine‐generated dimer (Fig. 5b). It may also be relevant that polyhistidine can form coordination complexes with certain divalent metal ions, for example Ni^2+^ and Cu^2+^, minute traces of which may be present in any biological solution and which, when complexed with polyhistidine, may mimic free Pb^2+^ (e.g. Fig. 3).

It remains unclear whether polyhistidine chaperones the monomeric RG‐II molecule before or after the latter has formed a borate mono‐ester. Figure 1(a) arbitrarily shows a polycation molecule chaperoning a boron‐free monomeric RG‐II molecule. However, it is also possible that the polycation first binds to monomeric RG‐II–B(OH)_2_^−^ (the product of the ‘fast’ reaction shown in Fig. 1a), aided by the additional charge on the borate group.

RG‐II was also rapidly dimerized by B(OH)_3_ in the presence of another polycation – spinach extensin. Since we routinely used 0.1 mg ml^−1^ monomeric RG‐II (*M*_r_ *c*. 5000) and 0.3 mg ml^−1^ spinach extensin (*M*_r_ *c*. 100 000), there was a *c*. 7 : 1 molar ratio of RG‐II : extensin. Some effect was also seen with 0.03 mg ml^−1^ extensin, that is, at a 70 : 1 molar ratio (or higher if the extensin was impure). Thus, with RG‐II + B(OH)_3_ as substrates, the extensin effect was clearly catalytic rather than stoichiometric. The possibility of an *in vivo* role for extensin in the boron bridging of RG‐II is compatible with the observation that members of the Poaceae (grasses and cereals) share three unusual features – a low pectin content (Albersheim *et al*., 2011), a low boron requirement for normal growth and development (Hu *et al*., 1996; Blevins & Lukaszewski, 1998), and a low extensin content (Lamport *et al*., 2011) – three parameters that would be expected to vary in parallel if RG‐II, boron and extensin act synergistically as we propose.

The extensin that is extractable from spinach cell cultures is a hydroxyproline‐rich basic glycoprotein with a high proportion of both lysine and histidine residues (7.8 and 6.3 mol%, respectively, of the total amino‐ plus imino‐acid residues), though with no detectable arginine (Biggs & Fry, 1990). It is interesting that the histidine content of different extensins varies widely (e.g. 1.0 mol% in tomato extensin ‘P2’, Smith *et al*., 1984; vs 11.4 mol% in carrot root disc extensin, Stuart & Varner, 1980), suggesting that this may be an important variable dictating the biological roles of different extensins. We propose that histidine‐rich extensins act as cationic molecular ‘chaperones’ of the RG‐II, enabling cross‐linking in a similar fashion to that achieved by Pb^2+^ or Sr^2+^, whereas histidine‐poor extensins may lack this function and instead serve different roles in plants. As with polyhistidine, the extensin evidently interacted with the RG‐II only transiently – catalytically rather than stoichiometrically, as the monomeric and dimeric RG‐II analysed after extensin treatment are electrophoretically indistinguishable from free monomer and the Pb^2+^‐generated dimer, respectively.

In the mature primary cell wall, a proportion of the extensin is likely to be ionically bonded to de‐esterified homogalacturonan, potentially limiting the extensin's availability for chaperoning RG‐II. However, such ionic bonding is unlikely to be irreversible. We note that the *in vitro* RG‐II–extensin interaction studied here is clearly reversible (see earlier), even though RG‐II has more anionic groups (namely the polyanionic backbone, the anionic side‐chain residues, and possibly already an esterified borate ester) than fully de‐esterified homogalacturonan (whose negative charges are contributed by the backbone only). Furthermore, during and shortly after pectin biosynthesis (i.e. when and where boron‐bridging is principally occurring; Chormova *et al*., 2014a,b), many of the homogalacturonan's GalA residues are methylesterified and therefore not anionic. Therefore, we do not regard homogalacturonan–extensin bonding as an impediment to the hypothesis that extensin can chaperone RG‐II and contribute to boron bridging.

Cation‐exchange chromatography of the crude spinach extensin preparation yielded a range of fractions each of which was moderately effective at promoting the dimerization of RG‐II. Thus, either several basic (glyco)proteins may share a limited ability to chaperone RG‐II and none was present at a sufficient concentration in the separated ion‐exchange fractions, or else a particularly effective but quantitatively minor glycoprotein may have been lost during chromatography.

### Conclusion

This work shows that certain polycations, including artificial polyhistidine and natural extensin, have the ability to chaperone RG‐II, manoeuvring this anionic polysaccharide domain so as to favour cross‐linking via boron bridges. A role for histidine‐rich extensins in cell wall assembly is therefore proposed. It is possible that extensins fulfilling this role are located on the inner face of the cell wall, ready to chaperone any new RG‐II domain as soon as it is secreted into the wall. Alternatively there could be sufficient extensin located inside the Golgi cisternae or Golgi‐derived vesicles to dimerize newly synthesized RG‐II domains even before secretion. Since both the polyhistidine and the proposed natural chaperone extensin can evidently dissociate from RG‐II after having facilitated its dimerization, we propose that these cationic chaperones act catalytically rather than stoichiometrically. In future research it will be of great interest to determine the ability of distinct isoforms of extensin, and other cationic glycoproteins, to direct the assembly of the wall's pectic network in this way.

## Supporting information

Please note: Wiley Blackwell are not responsible for the content or functionality of any supporting information supplied by the authors. Any queries (other than missing material) should be directed to the *New Phytologist* Central Office.

**Fig. S1** Some poly(amino acids) affect RG‐II dimerization.
