# Supplementary material for: Boron bridging of rhamnogalacturonan‐II is promoted in vitro by cationic chaperones, including polyhistidine and wall glycoproteins
Source: New Phytol. 2015 Aug 24;209(1):241–51. doi: 10.1111/nph.13596 (PMC4973674; doi:10.1111/nph.13596)
Supplement: Supplementary file 1 — Fig. S1 Some poly(amino acids) affect RG‐II dimerization. [file NPH-209-241-s001.pdf]

Supporting Information Fig. S1

Article title: Boron bridging of rhamnogalacturonan-II is promoted *in vitro* by cationic chaperones, including polyhistidine and wall glycoproteins

Authors: Dimitra Chormova and Stephen C. Fry

Article acceptance date: 25 June 2015

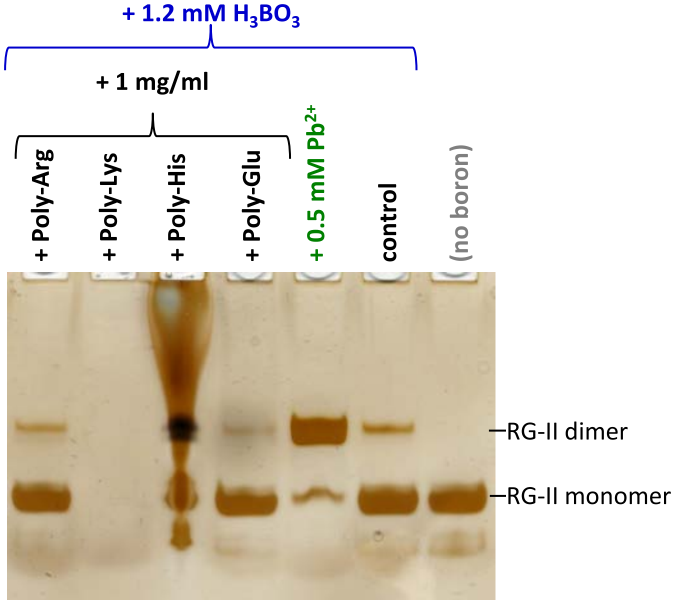

Fig. S1 Some polyamino acids affect RG-II dimerisation. Monomeric RG-II (0.1 mg ml^{-1}; 20 μM) was incubated for 24 h with the additives indicated. Other details as in Fig. 2.
